# Injury of the lateral vestibulospinal tract in a patient with the lateral medullary syndrome: Case report

**DOI:** 10.1097/MD.0000000000022117

**Published:** 2020-09-11

**Authors:** Sung Ho Jang, Ga Young Park, In Hee Cho, Sang Seok Yeo

**Affiliations:** a Department of Physical Medicine and Rehabilitation, College of Medicine, Yeungnam University, Gyeongsangbuk-do, South Korea; b Department of Health, Graduate School; c Department of Physical Therapy, College of Health Sciences, Dankook University, Chungnam, Republic of Korea.

**Keywords:** central vestibular disorder, diffusion tensor imaging, lateral medullary syndrome, vestibulospinal tract, Wallenberg syndrome

## Abstract

**Rationale:**

Lateral medullary syndrome is a central vestibular disorder characterized by vertigo and ataxia. We report on a patient with injury of the lateral vestibulospinal tract (VST) following lateral medullary syndrome, detected on diffusion tensor tractography (DTT).

**Patient concerns:**

A 56-year-old male patient was diagnosed with lateral medullary syndrome due to an infarction in the posterior inferior cerebellar artery area.

**Diagnoses:**

Two weeks following the infarction, he was transferred to the rehabilitation department of the same university hospital with severe vertigo, ataxia (Berg balance scale: 16 point), and dysphasia. In contrast, he maintained good motor power and cognitive function (Mini-mental state test: 26 points).

**Interventions:**

N/A

**Outcomes:**

Both the patient's medial VSTs and left lateral VST were well-reconstructed. In contrast, the right lateral VST was not reconstructed. On DTT parameters of the VST, the patient's medial VSTs and left lateral VST did not differ significantly from the control subjects.

**Lessons:**

An injury of the right lateral VST was demonstrated in a patient with lateral medullary syndrome. We believe that the result will be helpful in clinical management and research for patients with lateral medullary syndrome.

## 1 Introduction

Lateral medullary syndrome is a central vestibular disorder caused by infarction of the dorsolateral medulla due to stenosis of the posterior inferior cerebellar artery.^[1–4]^ The characteristic features of the lateral medullary syndrome are nystagmus, dysphasia, and disorder in vocalization, including dysarthria and hoarseness.^[1–6]^ Acute vertigo and ataxia are common sequelae of lateral medullary syndrome due to the vestibular nuclei lesions.^[1,5,6]^ The vestibular nuclei transmit motor commands for maintaining balance of upright posture of body and head through the vestibulospinal tract (VST) to spinal cord.^[6–13]^ Several clinical studies note the importance of the VST for balance and gait.^[5,6,8,12]^ Recent developments in diffusion tensor tractography (DTT), which is derived from diffusion tensor imaging (DTI), allow for visualization and localization of the medial and lateral VST in 3 dimensions.^[14]^ However, no study has reported injury of the VST following brain injury.

In this study, we report on a patient who injured the lateral VST following lateral medullary syndrome, detected on DTT.

## 2 Case presentation

One patient and six control subjects of similar age (3 males: mean age 56.7, range 51–58 years) with no history of neurologic disease participated in this study. All subjects provided signed, informed consent, and the study protocol was approved by the institutional review board of Yeungnam University.

**Figure 1:**
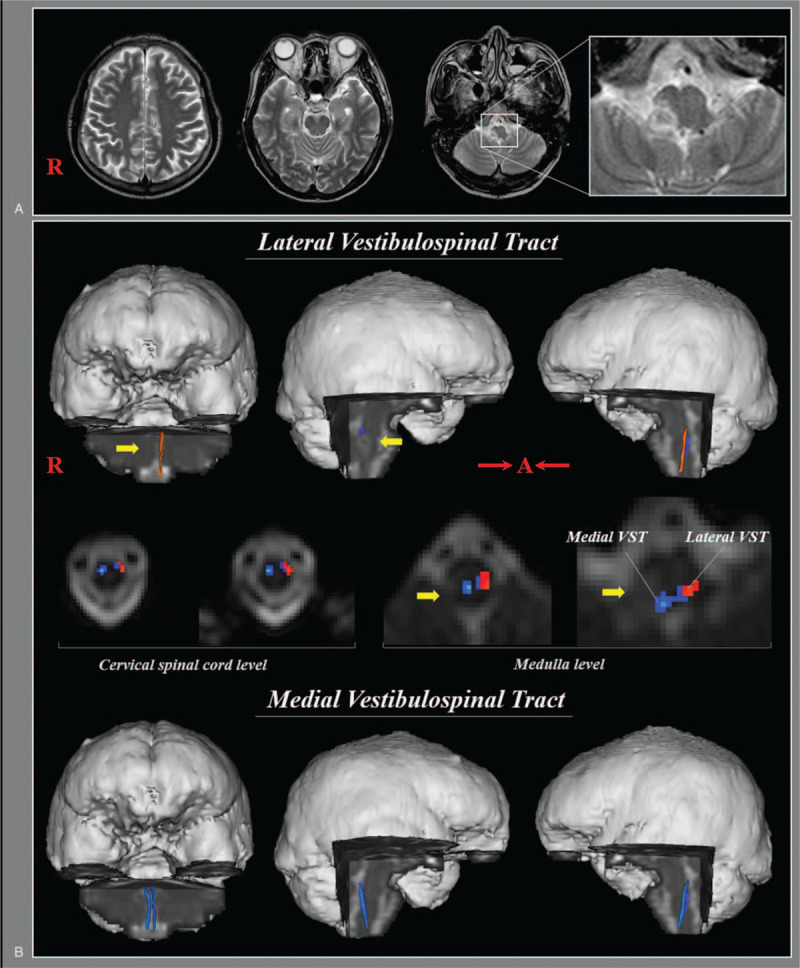
(A) Brain MR images at 2 weeks after onset show an infarction in the right dorsolateral medulla. (B) Results of diffusion tensor tractography (DTT). DTTs for both the medial vestibulospinal tract (VST) and the left lateral VST originate from the pontine vestibular nuclei and terminate at upper cervical cord. By contrast, DTT for the right lateral VST is not reconstructed between the lateral vestibular nuclei in pons and the upper cervical cord.

A 56-year-old male patient was diagnosed with lateral medullary syndrome due to an infarction in the posterior inferior cerebellar artery territory at the neurology department of a university hospital (Fig.1A). Two weeks following the infarction, he was transferred to the rehabilitation department of the same university hospital with severe vertigo, ataxia [Berg balance scale (BBS): 16 point and functional ambulation category (FAC): 2 grade], and dysphasia.^[15]^ In contrast, he maintained good motor power (Manual muscle test: good grade in upper and lower extremity) and cognitive function (Mini-mental state test: 26 points).^[16,17]^ In the evaluation after 6 weeks from on set, BBS scored 17 points and FAC scored 2 grade, and ataxia and gait problems persisted.

### 2.1 Diffusion tensor image

DTI data were acquired 2 weeks after the initial injury using a 6-channel head coil on a 1.5 T Philips Gyro scan Intera (Philips, Best, The Netherlands) and single-shot echo-planar imaging. For each of the 32 noncollinear diffusion sensitizing gradients, 67 contiguous slices were acquired parallel to the anterior commissure-posterior commissure line. Imaging parameters were as follows: acquisition matrix = 96 × 96; reconstructed matrix = 192 × 192; field of view = 240 × 240 mm^2^; TR = 10,726 ms; TE = 76 ms; parallel imaging reduction factor (SENSE factor) = 2; EPI factor = 49; b = 1000 s/mm^2^; NEX = 1; and a slice thickness of 2.5 mm with no gap (acquired voxel size 1.3 × 1.3 × 2.5 mm^3^).

### 2.2 Probabilistic fiber tracking

Diffusion-weighted imaging data were analyzed using the Oxford Centre for Functional Magnetic Resonance Imaging of the Brain (FMRIB) Software Library (FSL; www.fmrib.ox.ac.uk/fsl). Affine multiscale 2-dimensional registration was used for correction of head motion effect and image distortion due to eddy current. Fiber tracking was performed using a probabilistic tractography method based on a multifiber model, and applied in the present study utilizing tractography routines implemented in FMRIB Diffusion (5000 streamline samples, 0.5 mm step lengths, curvature thresholds = 0.2).

The medial VST was determined by selection of fibers passing through seed and 2 target regions of interest (ROI) (Fig. 1A). The medial VST is originated in the medial vestibular nuclei, Schwalbe nuclei, in the pons and medulla level, and terminated in the anterior funiculus of the cervical spinal cord.^[10,13,18]^ Therefore, we set the seed ROI at the medial vestibular nuclei in the caudal portion of the pons and the target ROI on the posteromedial medulla, corresponding to the medial vestibular nuclei in the medulla. The lateral VST originates in the Deiters’ nucleus or lateral vestibular nuclei of the pons, and descends through the reticular formation of medulla to lateral funiculus of spinal cord.^[10,13,18,19]^ Therefore, for analysis of the lateral VST, the seed ROI was placed on the lateral vestibular nuclei at the level of pons, and the target ROI on the posterolateral medulla, which corresponds to the reticular formation of the medulla. Out of 5000 samples generated from the seed voxel, results for contact were visualized threshold at a minimum of 1 streamline through each voxel for analysis. Values of fractional anisotropy (FA), mean diffusivity (MD), and tract volume of the medial and lateral VST were measured. DTI parameters showing a deviation of more than 2 standard deviations (SDs) of that of normal control values were defined as abnormal.

The reconstructed medial VST in both hemispheres originated from the pontine medial vestibular nuclei and terminated at the anterior funiculus of the spinal cord. The left lateral VST in the unaffected side was reconstructed between the pontine lateral vestibular nuclei and lateral funiculus of spinal cord. In contrast, the right lateral VST in the affected side was not reconstructed (Fig. 1B). The patient's medial VST did not differ significantly from control subjects, nor did DTT parameters of the left lateral VST (Table 1).

**Table 1:**
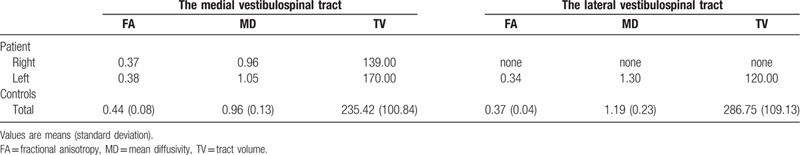
Comparison of diffusion tensor parameters of the medial and lateral vestibulospinal tract between a patient and control group.

## 3 Discussion

Using DTT, we investigated an injury of the lateral VST in a patient with lateral medullary syndrome. Both the medial VSTs and the left lateral VST were well-reconstructed between pontine vestibular nuclei and spinal cord. In addition, the FA, MD, and tract volume of both medial VST and left lateral VST did not differ significantly compared with those of the control group. The FA value represents the degree of directionality of microstructures, and the MD value indicates the magnitude of water diffusion in tissue, which can increase with some forms of pathology or neuronal injury.^[20,21]^ The tract volume is determined by the number of voxels contained within a neural tract.^[20,21]^ Therefore, no significant differences in these DTT parameters in both the medial VST and the left VST imply that there was no neuronal injury after the dorsolateral medullary infarction. By contrast, the non-reconstruction of the right lateral VST in the affected side indicate severe neural injury from the right dorsolateral medullary infarct. The severe vertigo and ataxia in the patient appeared to be mainly attributable to the injury of the right lateral VST.

A few studies describe vestibular-evoked myogenic potential (VEMP), using vestibular nerve stimulation at the mastoid level, as a diagnostic method for the patient with lateral medullary syndrome.^[22,23]^ In 2008, Lundy et al^[22]^ reported a patient with dizziness, double vision, hoarseness, and dysphagia after dorsolateral medullary infarction. The patient had abnormally enlarged VEMP results, associated with labyrinthine hydromechanical changes.^[22]^ In 2010, Tseng and Young^[23]^ reported on the topographical correlations of lateral medullary infarction with caloric and VEMP results. All 5 patients developed vertigo, vomiting, and ataxia after lateral medullary infarction. However, caloric test and VEMP test were both abnormal at 60%.^[23]^ To the best of our knowledge, there has been only one neuroimaging study of the pathological etiology of lateral medullary syndrome.^[4]^ In 2005, using positron emission tomography (PET), Diethrich et al^[4]^ reported changes of cortical activation by caloric vestibular stimulation in patients with lateral medullary syndrome.^[4]^ They suggested that in all 3 patients, the crossing fibers between the medial vestibular subnucleus and the contralateral medial longitudinal fascicle were affected. During caloric vestibular stimulation condition, all patients showed decreased activation in the contra-lesional hemisphere, compared with normal control subjects.

As far as we are aware, this is the first DTI study to identify injury to the lateral VST in a patient with sever lateral medullary syndrome. However, several limitations of this study should be considered. First, because it is a case report, the results of this study are descriptive in nature. Second, DTI may underestimate fiber tracts, and regions of fiber complexity and crossing can prevent full reflection of the underlying fiber architecture by DTI.^[24]^ Last, we could not precisely set the location of ROIs because of cramped size of vestibular nuclei.

In conclusion, injury to the right lateral VST was detected in a patient with lateral medullary syndrome. We believe that this study will be helpful in clinical diagnosis, management, and research for patients with lateral medullary syndrome due to the injury of VST. Further studies involving larger case numbers are warranted and more detailed clinical correlation with injury of the VST will be needed in the near future.

## Author contributions

**Conceptualization:** Sung Ho Jang, Sang Seok Yeo

**Data curation:** Sung Ho Jang, Ga Young Park, In Hee Cho, Sang Seok Yeo

**Formal analysis:** Sung Ho Jang, Sang Seok Yeo

**Funding acquisition:** Sang Seok Yeo

**Investigation:** Sung Ho Jang, Ga Young Park, In Hee Cho, Sang Seok Yeo

**Methodology:** Sung Ho Jang, Ga Young Park, In Hee Cho, Sang Seok Yeo

**Writing – original draft:** Ga Young Park, In Hee Cho

**Writing – review & editing:** Sung Ho Jang, Sang Seok Yeo

## References

[1] Waespe W Zahner S Acute vestibular syndrome in cerebellar infarct of the posterior inferior cerebellar artery (PICA infarct). Schweiz Med Wochenschr 1996;126:214 9. 8720725

[2] Dieterich M Brandt T Imaging cortical activity after vestibular lesions. Restor Neurol Neurosci 2010;28:47 56. 20086282 10.3233/RNN-2010-0505

[3] Day GS Swartz RH Chenkin J Lateral medullary syndrome: a diagnostic approach illustrated through case presentation and literature review. CJEM 2014;16:164 70. 24626124 10.2310/8000.2013.131059

[4] Dieterich M Bense S Stephan T Medial vestibular nucleus lesions in Wallenberg's syndrome cause decreased activity of the contralateral vestibular cortex. Ann N Y Acad Sci 2005;1039:368 83. 15826990 10.1196/annals.1325.035

[5] Na EH Yoon TS Han SJ Improvement of quiet standing balance in patients with wallenberg syndrome after rehabilitation. Ann Rehabil Med 2011;35:791 7. 22506207 10.5535/arm.2011.35.6.791 PMC3309380

[6] Jung JH Yoo MH Song CI Prognostic significance of vestibulospinal abnormalities in patients with vestibular migraine. Otol Neurotol 2015;36:282 8. 25369908 10.1097/MAO.0000000000000656

[7] Highstein SM Holstein GR The anatomical and physiological framework for vestibular prostheses. Anat Rec (Hoboken) 2012;295:2000 9. 23044714 10.1002/ar.22582 PMC4039022

[8] Zorner B Bachmann LC Filli L Chasing central nervous system plasticity: the brainstem's contribution to locomotor recovery in rats with spinal cord injury. Brain 2014;137:1716 32. 24736305 10.1093/brain/awu078

[9] Lambert FM Bras H Cardoit L Early postnatal maturation in vestibulospinal pathways involved in neck and forelimb motor control. Dev Neurobiol 2016;76:1061 77. 26724676 10.1002/dneu.22375

[10] Adel K Afifi RAB Functional Neuroanatomy: Text and Atlas. 2nd ed. New York: McGraw-Hill; 2005.

[11] Green AM Angelaki DE Internal models and neural computation in the vestibular system. Exp Brain Res 2010;200:197 222. 19937232 10.1007/s00221-009-2054-4 PMC2853943

[12] Markham CH Vestibular control of muscular tone and posture. Can J Neurol Sci 1987;14: 3 suppl: 493 6. 3315150 10.1017/s0317167100037975

[13] Sadjadpour K Brodal A The vestibular nuclei in man. A morphological study in the light of experimental findings in the cat. J Hirnforsch 1968;10:299 323. 5732949

[14] Jang SH Kwon JW Yeo SS Three dimensional identification of medial and lateral vestibulospinal tract in the human brain: a diffusion tensor imaging study. Front Hum Neurosci 2018;12:229. 29922138 10.3389/fnhum.2018.00229 PMC5996120

[15] Downs S Marquez J Chiarelli P The Berg Balance Scale has high intra- and inter-rater reliability but absolute reliability varies across the scale: a systematic review. J Physiother 2013;59:93 9. 23663794 10.1016/S1836-9553(13)70161-9

[16] Florence JM Pandya S King WM Intrarater reliability of manual muscle test (Medical Research Council scale) grades in Duchenne's muscular dystrophy. Phys Ther 1992;72:115 22. discussion 122-126. 1549632 10.1093/ptj/72.2.115

[17] Jeong SK Cho KH Kim JM The usefulness of the Korean version of modified Mini-Mental State Examination (K-mMMSE) for dementia screening in community dwelling elderly people. BMC Public Health 2004;4:31. 15283869 10.1186/1471-2458-4-31 PMC509251

[18] Nathan PW Smith M Deacon P Vestibulospinal, reticulospinal and descending propriospinal nerve fibres in man. Brain 1996;119:1809 33. 9009990 10.1093/brain/119.6.1809

[19] Vernon W Lin DDC Spinal Cord Medicine: Principles and Practice. 2nd ed. New York: Demos Medical; 2010.

[20] Assaf Y Pasternak O Diffusion tensor imaging (DTI)-based white matter mapping in brain research: a review. J Mol Neurosci 2008;34:51 61. 18157658 10.1007/s12031-007-0029-0

[21] Mori S Crain BJ Chacko VP Three-dimensional tracking of axonal projections in the brain by magnetic resonance imaging. Ann Neurol 1999;45:265 9. 9989633 10.1002/1531-8249(199902)45:2<265::aid-ana21>3.0.co;2-3

[22] Lundy L Zapala D Olsholt K Dorsolateral medullary infarction: a neurogenic cause of a contralateral, large-amplitude vestibular evoked myogenic potential. J Am Acad Audiol 2008;19:246 56. quiz 275. 18672653 10.3766/jaaa.19.3.9

[23] Tseng CL Young YH Topographical correlations of lateral medullary infarction with caloric- and vestibular-evoked myogenic potential results. Eur Arch Otorhinolaryngol 2010;267:191 5. 19562359 10.1007/s00405-009-1025-5

[24] Yamada K Sakai K Akazawa K MR tractography: a review of its clinical applications. Magn Reson Med Sci 2009;8:165 74. 20035125 10.2463/mrms.8.165

